# Influence of Mechanical Stimuli on Schwann Cell Biology

**DOI:** 10.3389/fncel.2017.00347

**Published:** 2017-11-01

**Authors:** Sophie Belin, Kristen L. Zuloaga, Yannick Poitelon

**Affiliations:** Department of Neuroscience and Experimental Therapeutics, Albany Medical College, Albany, NY, United States

**Keywords:** Schwann cell, mechanobiology, mechanotransducer, mechanosensor, myelin, peripheral nerve

## Abstract

Schwann cells are the glial cells of the peripheral nervous system (PNS). They insulate axons by forming a specialized extension of plasma membrane called the myelin sheath. The formation of myelin is essential for the rapid saltatory propagation of action potentials and to maintain the integrity of axons. Although both axonal and extracellular matrix (ECM) signals are necessary for myelination to occur, the cellular and molecular mechanisms regulating myelination continue to be elucidated. Schwann cells in peripheral nerves are physiologically exposed to mechanical stresses (i.e., tensile, compressive and shear strains), occurring during development, adulthood and injuries. In addition, there is a growing body of evidences that Schwann cells are sensitive to the stiffness of their environment. In this review, we detail the mechanical constraints of Schwann cells and peripheral nerves. We explore the regulation of Schwann cell signaling pathways in response to mechanical stimulation. Finally, we provide a comprehensive overview of the experimental studies addressing the mechanobiology of Schwann cells. Understanding which mechanical properties can interfere with the cellular and molecular biology of Schwann cell during development, myelination and following injuries opens new insights in the regulation of PNS development and treatment approaches in peripheral neuropathies.

## Introduction

Peripheral nerves are remarkable tissues of tremendous elasticity that propagate action potentials despite developmental growth (Vizoso and Young, 1948; Court et al., 2004; Simpson et al., 2013), stretches associated with movements of the limbs and mechanical compressions from daily activities (Kwan et al., 1992; Phillips et al., 2004). To resist mechanical stress, peripheral nerves are supported by three layers of connective tissues (i.e., epineurium, perineurium and endoneurium). In addition, each axon is individually wrapped by a large extension of modified plasma membrane called myelin sheath. Myelin appeared initially in hinged jaw fishes and was carried out in cartilaginous fishes. It contributed to the evolutionary success of larger vertebrates by allowing a faster and efficient conduction of nerves action potentials (Morell and Quarles, 1999), thus enabling faster predatory and escape maneuvers and the development of large and complex nervous systems (Zalc and Colman, 2000; Hartline and Colman, 2007; Zalc et al., 2008). In addition, to enhance the conduction of electric impulses and protect axons from mechanical stress damage, myelin producing cells also support the exchange of metabolites between the regional vasculature and the axon (Viader et al., 2011; Fünfschilling et al., 2012; Lee et al., 2012).

**Figure 1:**
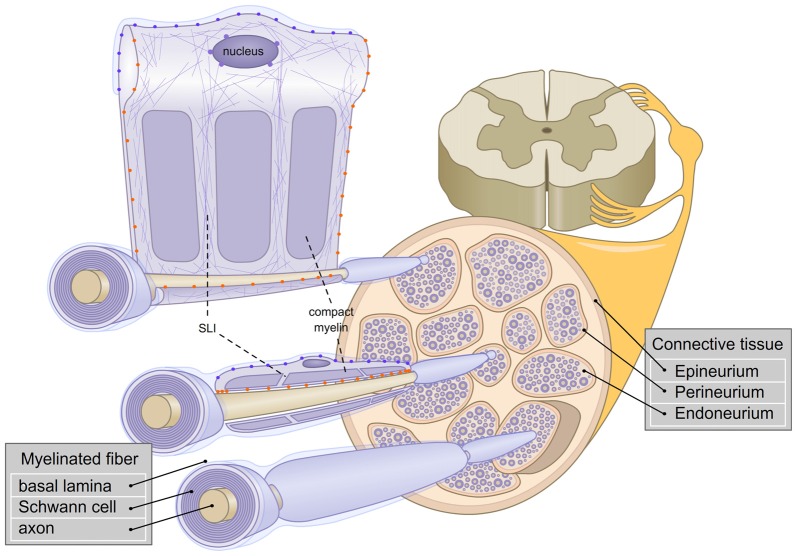
Schematic representation of the architecture of myelinated fibers in the peripheral nervous system (PNS). The three layers of connectives tissues are depicted and three myelinated fibers are enlarged. The first fiber (bottom) represents a rolled Schwann cell (SC). The second fiber (middle) shows a longitudinal section of a Schwann cell. The third fiber (top), is a view of Schwann cell unrolled. Axonal (orange 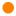) and basal lamina (blue 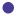) cell adhesion molecules (CAMs) and Linker of Nucleoskeleton and Cytoskeleton complex (LINC; purple 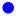) are represented. Actin filaments are depicted with purple thin lines. SLI: Schmidt-Lanterman incisures.

In the peripheral nervous system (PNS), Schwann cells (SCs) produce the myelin sheath. They are among the largest known cells, with 0.6 mm^2^ of membrane on average (Rosenbluth, 2005). Understanding myelinating SC architecture can be challenging. To initiate myelination, SCs require an apicobasal polarity created by the axon (apical side) and the extracellular matrix (ECM; basal side). In addition, each myelin segment is flanked by nodes of Ranvier; thus creating a remarkable longitudinal polarity with internodal, juxtaparanodal and paranodal regions (Masaki, 2012; Figure 1). Yet, regardless of their gigantic myelin sheath and complex ultrastructure, SCs have a surprising plasticity. Upon injury to peripheral nerves, myelinated SCs can dedifferentiate and drive the regeneration of peripheral axons (Jessen and Mirsky, 2016; Boerboom et al., 2017). Outstanding reviews on SC development and the regulation of myelination in the PNS have recently been published and may be consulted for comprehensive characterization of SC biology (Kidd et al., 2013; Feltri et al., 2015; Monk et al., 2015; Salzer, 2015). In this review, we will focus on what the environmental constraints are for SCs, how SCs can sense and transduce mechanical signals and the experimental approaches that have started to unveil SC mechanobiology.

## Peripheral Nerves and Schwann Cells Resistance to Mechanical Stresses

Peripheral nerves and SCs have adapted to sustain constant mechanical constraints. In peripheral nerves, myelinated fibers are surrounded by 6–15 layers of connective tissues (Sunderland, 1990) which shield SCs and axons from strains coming from the external environment (Lundborg and Rydevik, 1973; Rydevik et al., 1990; Wall et al., 1991, 1992; Kwan et al., 1992; Brown et al., 1993). As previously mentioned, the connective tissue in peripheral nerves is constituted of three layers: the epineurium, the perineurium and the endoneurium. The epineurium constitutes a thick layer which surrounds the nerve and contributes to the resilience of the nerve (Peltonen et al., 2013). The perineurium is a strong layer of epithelium-like cells and collagen fibers (Thomas and Jones, 1967). Finally, the endoneurial ECM is found near the myelinated fibers, to protect them further from mechanical stresses. Interestingly, the endoneurial fluid is at a higher pressure than the epineurial fluid. An additional role for the perineurium could be to withstand the positive pressure from the endoneurium (Myers et al., 1978; Figure 1).

The stiffness and elasticity of peripheral nerves determine the mechanical cues that SCs are exposed to. They are partially determined by the connective tissue surrounding the nerves. Yet the elastic properties of peripheral nerves are not equal along their length. It is increased near articulations where nerves need to be more elastic, which reveals a more complex tissue architecture than anticipated (Phillips et al., 2004). For many years researchers have observed the undulating course of peripheral nerve fibers, also called spiral bands of Fontana (Zachary et al., 1993). Bands of Fontana found in peripheral nerves disappear when nerves are stretched by 10% of their original length (Pourmand et al., 1994). Thus, bands of Fontana are thought to promote peripheral nerve elasticity. In support of this hypothesis, bands of Fontana are absent from nerves not subject to mechanical strain, such as newly regenerated nerves, intracranial nerves or spinal roots (Clarke and Bearn, 1972; Merolli et al., 2012). The mechanism by which bands of Fontana are formed is still unclear (Power et al., 2015).

SCs themselves adapted to endure external mechanical forces. Most of the SC myelin segment consists of compact myelin. Yet, compact myelin is interrupted by regions of uncompact myelin called Schmidt-Lanterman incisures (Small et al., 1987). Thus, it was proposed that incisures are a reservoir of membrane, allowing SCs to respond to a stretch of the myelin segment (Glees, 1943). While Schmidt-Lanterman incisures also have a role to play in SC intracellular transport (Balice-Gordon et al., 1998), it is important to point out that they are found only in the PNS, and their presence is conditional to the expression of P0, a major peripheral myelin protein (Yin et al., 2008, 2015). SCs also contributes to the mechanical resistance of peripheral nerves by secreting the basal lamina. The basal lamina is an essential component of the SC ECM which is critical for SC development, surrounding each myelinated fiber (Feltri et al., 2015; Monk et al., 2015) and is an important component allowing regeneration of the peripheral nerves. The basal lamina organization remains unaffected after a severe mechanical compression on the nerve, or an acute axonal degeneration and is even further maintained after axonal death. As such, the basal lamina is able to be used as a guidance for axonal regrowth (Bunge et al., 1989). More recent methods have confirmed that the basal lamina provides a mechanical resistance to compression (Rosso et al., 2014). Autotypic junctions of SCs, formed between the spiral layers of myelin, have also been hinted to contribute to SC mechanical strength (Poliak et al., 2002). However, several models have shown that disruption of SC autotypic junctions impairs the propagation of action potentials but does not affect the appearance of the myelin sheath (Miyamoto et al., 2005; Denninger et al., 2015). Taken together, these studies suggest that relative elasticity or stiffness of the peripheral nerves influence the mechanical cues that the SCs are exposed to, while SC architecture and basal lamina integrity play key roles in the SC response to mechanical stress.

## Mechanical Injuries to Peripheral Nerves and Consequence to Schwann Cell Biology

**Figure 2:**
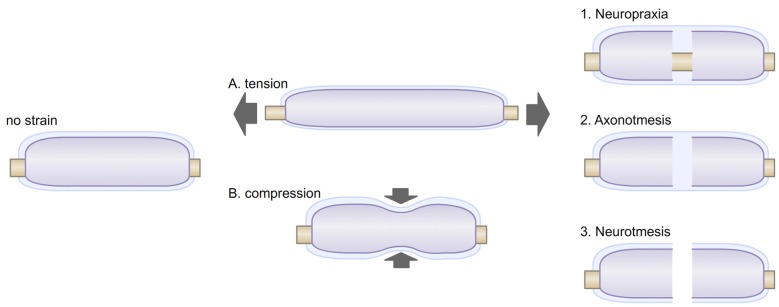
Myelinated fibers may be exposed to different stresses. **(A)** Tension strain can be caused by postures or movements. Under tension, the diameter of the myelinated fiber is also reduced, creating a transverse contraction strain. **(B)** Compression strain can be created by neighboring tissues (muscle, tendon, bone) or by environmental strains. External compression of peripheral nerves has been shown to alter myelin paranodal organization and can lead to demyelination (Dyck et al., 1990; Hodgson, 1993). Exposures to these stresses can caused various type of injuries, i.e., neuropraxia (1), in which myelin can be disrupted; axonotmesis (2), in which axons are disrupted; neurotmesis (3), in which the whole myelinated fiber, including its basal lamina are disrupted.

The pathological consequences of mechanical peripheral nerves injuries are evident, but their severity can vary. These injuries, were first classified by Seddon et al. (1943) ranked in three degrees: neurotmesis, axonotmesis and neuropraxia. Neurotmesis are dramatic injuries in which axons, connective tissues and basal lamina deposition are disconnected (Figure 2). They cause a complete loss of function. The recovery after neurotmesis is poor. Therapeutic approaches taking advantage of nerve grafts or nerve conduits have been attempted, but the success of such approaches have been mixed with variable improvements in functional recovery (Battiston et al., 2005). On the other hand, axonotmesis are severe compressions of the nerve or stretch-related injuries, and cause a disruption of axons and myelin sheaths with preservation of connective tissues and basal lamina (Figure 2). Distally to the point of trauma, axons degenerate and SCs demyelinate. Within 30 days after injury, peripheral nerves can remarkably recover, with axon regeneration and SC remyelination. Finally, in neuropraxia peripheral nerves endure more common and less severe trauma that can be caused by mechanical compression of the nerve or ischemia (Ochoa et al., 1971, 1972). In neuropraxia, axon integrity is not primarily disrupted, but demyelination can occur (Figure 2). This injury affects the propagation of action potential, but is fully reversible, as the “Saturday night palsy” (Sunderland, 1990; Lee and Wolfe, 2000). Chronic compressions are another form of injury in peripheral nerves (Brown et al., 2011). They start with the inability of axons to transmit action potentials and as compression become more severe; demyelination follows (Rempel et al., 1999; Burnett and Zager, 2004). The effect of chronic nerve compression has been well characterized (Mackinnon et al., 1985; O’Brien et al., 1987; Dahlin et al., 1993; Pham et al., 2009). Chronic mechanical stimulation induces SC demyelination, as well as an increase in both SC apoptosis and proliferation, in absence of evident axonal injury (Gupta and Steward, 2003; Gupta et al., 2012). Similarly *in vitro* shear stress on primary SCs in the form of a laminar fluid flow is sufficient to reduce myelin protein gene expression and promote SC proliferation (Gupta and Steward, 2003; Gupta et al., 2005). Thus, low level mechanical stimuli may activate SC mitogenic pathways, independently from the regulation occurring between SCs and axons after axonal injury (Salzer and Bunge, 1980).

It is also worth mentioning that genetic neuropathies could also be related to mechanical stresses. This appears to be the case with the hereditary neuropathy with liability to pressure palsies (HNPP). In this disease caused by the haploinsufficency of *PMP22*, patients are challenged daily by everyday activities. Benign mechanical pressures like crossing the legs can cause weakness or paralysis in the foot for several hours to several months (Earl et al., 1964; Horowitz et al., 2004; Li, 2015). The causes of HNPP are unclear as the exact role and functions of PMP22 are still being deciphered. Yet, a notable observation in HNPP myelinated fibers is the disruption of SCs autotypic junctions (Guo et al., 2014; Hu et al., 2016). Thus, it is possible that in SCs haploinsufficient for PMP22, the myelin is less resilient to mechanical stresses. This hypothesis is supported by atomic force microscopy work on myelinated fibers knock-out for PMP22 (Rosso et al., 2014). Thus, the response of SCs to mechanical stresses plays a role in both traumatic neuropathies and some genetic neuropathies.

## Extracellular and Intracellular Components of SC Mechanobiology

Mechanobiology refers to how physical and mechanical signals can be sensed by organs, tissues or cells and are converted in specific cellular responses. Many fundamental aspects of cell behavior depend on mechanobiology, including adhesion, spreading, migration, gene expression and cell-cell interactions in multiple cell-types (Jansen et al., 2015). Mechanobiology is based on the existence of two components: (1) mechanosensors, that allow a cell to sense mechanical signals provided by its environment; and (2) mechanotransducers, that allow a cell to transduce mechanical signals into biochemical signals. A large amount of mechanosensors and mechanotransducers from both intra and extracellular compartments have been identified over the last decades (Jansen et al., 2015). However these components are not relevant to all cells (e.g., ciliary bundles on hair cells in the sensory macula, glycocalix presence at the plasma membrane of epithelial cells, etc.; Jaalouk and Lammerding, 2009) and it is still unclear how mechanosensation and -transduction are orchestrated. In SCs, the identity of mechanotransducers and mechanosensors is starting to emerge. In the following section, we will review how physical signals can be transmitted in SCs through ECM, cell adhesion molecules (CAMs) and internal structures such as cortical cytoskeleton and the nucleus.

### Extracellular Matrix

ECM acts as a physical support for cells to allow their adhesion and migration, and for tissue to protect their architecture from mechanical stresses. Its composition includes hundreds of proteins (i.e., elastin and collagen), glycoproteins and numerous proteoglycans. In addition, like epithelial cells, SCs secrete a basal lamina, an ECM component composed of laminin and collagen networks cross-linked with proteoglycans and nidogens, which links the extracellular environment to the plasma membrane of SCs (Bunge et al., 1980). The mechanical properties of the ECM are defined mainly by elastic fibers and collagens fibers, which provide respectively resilience (elasticity) and tensile strength (resistance to tensile stress). In peripheral nerves, the proportion of elastic fibers in peripheral nerves is relatively small in comparison to collagen (Sunderland, 1965). In addition, collagen fibers are arranged longitudinally to allow some degree of axial stretch. Thus it was suggested that collagen fibers also contribute to the elastic properties of peripheral nerves (Tassler et al., 1994). Since all Schwann cells are surrounded by ECM, the most intuitive example of mechanotransduction is a transmission of signals through the ECM and the SC basal side. Yet, SCs also have an intimate relationship with axons on their apical side where biochemical signals from neurons are critical for SC migration, proliferation, survival, polarization, differentiation and gene expression (Monk et al., 2015; Salzer, 2015). For example, the myelinating fate of a SC is dictated by the amount of type III neuregulin-1 present on the surface of an axon (Taveggia et al., 2005). However, only membrane-bound type III neuregulin-1 promotes SC differentiation. In addition, a direct axonal contact is required for the deposition of a basal lamina by SCs (Bunge et al., 1982; Poitelon et al., 2015). These reports illustrate the functional need of SC to establish a direct physical contact with axons. Thus, SC fate depends on the formation of adhesion complexes between SCs with both axons and ECM. These adhesion complexes are primary sites of transduction for mechanical signals occurring in SCs and can be considered as mechanosensors.

### Cell Adhesion Molecules

**Figure 3:**
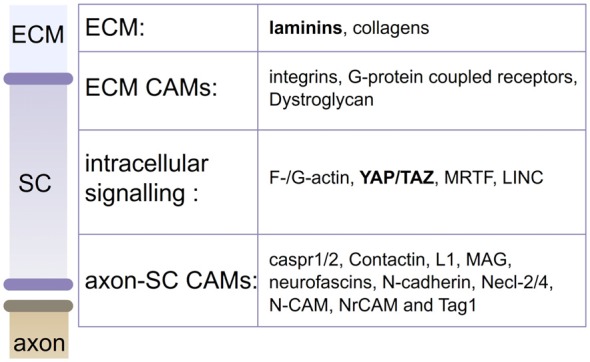
Actors in SC mechanobiology. Mechanical stimuli from the extracellular matrix (ECM; laminins and collagens) or the axon can be sensed by CAMs or actin cytoskeleton. Stimuli are then transduced into biological responses, by YAP/TAZ, MRTF or LINC. Laminins and YAP/TAZ (in bold) are confirmed contributors of SC mechanobiology (Poitelon et al., 2016).

Major adhesion complexes are formed by CAMs and are implicated at various stages of SC development (Poliak and Peles, 2003; Feltri and Wrabetz, 2005; Figure 3). Regarding their role as mechanosensors, SC CAMs can be divided into two different groups based on their interaction with either the axon or the ECM (Figure 1). Each myelinating SC extends up to 1 mm in length along an axon, and keeps a minimal gap between the two at about 15 nm (Abe et al., 2004; Salzer et al., 2008). To maintain this proximity, numerous CAMs are expressed on both SCs and axons, including Caspr1, Caspr2, Contactin, Dystroglycan, Integrin β1, L1, MAG, Neurofascin 155 and 186, N-cadherin, Necl-2, Necl-4, N-CAM, NrCAM and Tag1 (Salzer et al., 2008; Berti et al., 2011; Colombelli et al., 2015). These CAMs are critical for the tight apposition and polarized organization of SCs, for review, see (Beirowski, 2013). Genetic studies have shown that ablation of several of these proteins leads to impairments of the interaction between the SC and the axon or its maintenance (Feltri et al., 2015). In addition, stretch induced injuries could create a conduction block, caused presumably by the shear between axons and SCs at the nodes of Ranvier/paranodal region (Maxwell et al., 1991; Jou et al., 2000; Ichimura et al., 2005). The second group of CAMs, which distinctively interacts with the ECM, includes basal lamina receptors (i.e., integrin, G-protein coupled receptors and Dystroglycan). The role of these molecules has been well characterized during the last decades. SCs lacking basal lamina receptors experience detachment from the basal lamina and develop arrest or delay in the establishment of the interaction with an axon and its subsequent myelination (Feltri et al., 2015; Monk et al., 2015). Among these receptors, the role of integrin-mediated adhesions in mechanotransduction has been quite studied (Humphrey et al., 2014), and while some integrin-ECM interactions weaken when subjected to tension strain, often these adhesions strengthen or stabilize (Friedland et al., 2009; Litvinov et al., 2011). In particular, some β1 integrins have shown to strengthen under tension (Friedland et al., 2009; Kong et al., 2009). The behavior of SC α3β1, α6β1, α7β1 and α6β4 integrins under strain has not been defined yet (Berti et al., 2006), but is of interest as β1 integrins are essential for SC polarization and proper axonal ensheathment (Feltri et al., 2002).

Integrins are central to mechanosensation as they are connected both to the ECM and the actomyosin cytoskeleton, in clusters called focal adhesions. The structure of focal adhesions is complex and constitutes of numerous proteins. Thus, it is difficult to integrate the contribution of each one during mechanosensation. However, possible mechanically-induced stimulation of the focal adhesion complexes, could regulate important signaling pathways for SC biology, (i.e., downstream of FAK or ILK; Grove et al., 2007; Pereira et al., 2009; Grove and Brophy, 2014; Love et al., 2017), and stimulate the actin cytoskeleton in SCs. Indeed, Rac1 acts downstream of integrin β1 to drive actomyosin activity (Benninger et al., 2007; Nodari et al., 2007). The important role of actin in SC myelination was shown through the deletion of N-Wasp and Profilin1, regulators of actin polymerization (Jin et al., 2011; Novak et al., 2011; Montani et al., 2014). Yet, filaments of actin, linked to focal adhesions, also play a direct role in mechanosensation. The application of tensile force to cells increases their F-/G-actin ratio and promotes the formation of actomyosin filaments. In addition to the actin assembly, activation of myosin II or myosin light chain kinase is critical for SC myelination (Fernandez-Valle et al., 1997; Melendez-Vasquez et al., 2004; Wang et al., 2008; Leitman et al., 2011; Montani et al., 2014). Thus, integrins can affect SC mechanobiology via their actions as mechanosensors and via changes in actomyosin activity.

### Mechanotransduction Pathways

Although incompletely understood, the responses of the actomyosin cytoskeleton together with the activation of focal adhesion signaling induce several mechanotransducing pathways. The most notables are YAP and TAZ, two transcriptional activators of the Hippo pathway. Indeed, several works have shown that YAP can be activated through the Hippo pathway in SCs by Crb/Amolt proteins (Colciago et al., 2015; Fernando et al., 2016). In addition, mechanical stimulation through a signaling cascade involving FAK, Src, PI3K, JNK pathways (Codelia et al., 2014; Mohseni et al., 2014; Kim and Gumbiner, 2015; Elbediwy et al., 2016), or formation of actomyosin filaments and presence of F-actin (Dupont et al., 2011; Aragona et al., 2013) can regulate YAP and TAZ. Upon stimulation, YAP and TAZ are translocated to the nucleus to regulate gene transcription. In addition to regulating YAP and TAZ, F-/G-actin ratio also regulates the translocation of myocardin-related transcription factors (MRTFs) from the cytosol into the nucleus to activate serum response factor (SRF)-dependent transcription (Olson and Nordheim, 2010). While YAP/TAZ and MRTF transcriptional targets overlap (Esnault et al., 2014), MRTFs have also been shown to be upstream of YAP and TAZ and to facilitate the YAP/TAZ response (Cui et al., 2015). Mechanotransduction by YAP and TAZ is critical to SC development and will be discussed in depth later in this review; however, the functions of MRTFs in the peculiar architecture of SC remain to be explored. The application of forces, including high density plating and mechanical compression, acts on the actin cytoskeleton and can lead to deformation of the nucleus and influence chromatin organization (Hernandez et al., 2016). The Linker of Nucleoskeleton and Cytoskeleton complex (LINC) binds the nuclear envelope and the actin cytoskeleton fulfilling a role of mechanotransducer between the cell inner membrane and its nucleus (Baarlink et al., 2013; Isermann and Lammerding, 2013; Plessner et al., 2015). Epigenetic modulators of chromatin structures, such as histone deacetylases, have been shown to be extremely important for SC myelination (Chen et al., 2011; Jacob et al., 2011; Brügger et al., 2015; Wu et al., 2016), but the exact function of LINC in SC development is unclear.

### Mechanosensitive Ion Channels

Although much emphasis has been placed on cell adhesion complexes as force sensors, mechanosensitive ion channels contribute both to mechanosensation and transduction. Over the past few years, a number of advances have been made through the identification of new ion channels, expressed in nearly all cell types, such as mechanosensitive potassium channels and Piezo ion channels (Coste et al., 2010; Ranade et al., 2015). They showed that conformational changes in the lipid bilayer of the plasma membrane, such as shear or membrane curvatures, can be converted directly in electric or biochemical signals (Brohawn et al., 2014). This is particularly of interest in the SC, which function is to wrap their membrane around an axon. Interestingly, the importance of membrane curvature during myelination has already been suggested in previous works, which have highlighted the role of lipids in plasma membrane (Ohler et al., 2004; Shaharabani et al., 2016) or N-Wasp (Novak et al., 2011). Considering that SCs express ion channels (Gray et al., 1984; Barres et al., 1990), the role of potential mechanosensitive ion channels in SC should be considered. In summary, SC mechanobiology can be influenced by both extracellular and intracellular cues. Particularly important regulators are the ECM, CAMs, YAP/TAZ signaling and the cortical cytoskeleton.

## Experimental Approaches to SC Mechanobiology

Experimental approaches to study SC mechanobiology need to account for a variety of factors to model accurately the *in vivo* environment of peripheral nerves. During adult life, peripheral nerves are constantly subjected to mechanical stresses. In addition, the elasticity of peripheral nerves varies greatly along development, from 6 kPa after birth, when myelination is starting, to 50 kPa in adult nerves (Urbanski et al., 2016). This prompted the engineering community to investigate the response of SCs to their environment, in order to improve the design of nerve conduits used after injury (Gu et al., 2012; López-Fagundo et al., 2014). Isolated SCs grown on soft substrates (from 0.5 to 4 kPa) are rounded and present a low motility and a low proliferation rate (Gu et al., 2012; Poitelon et al., 2016). On stiffer substrates (≥40 kPa), SCs become bipolar and have an increased motility and proliferation (Gu et al., 2012; Poitelon et al., 2016). Surprisingly, SCs appear to be relatively insensitive to variations in stiff substrates, showing slight variations in morphology and no variations in the activation in YAP/TAZ mechanotransducers from 40 kPa to 4 MPa (Poitelon et al., 2016). However, when substrates are enriched with laminin, SC response is vastly different. On soft substrate coated with laminin (around 1 kPa), SCs are able to maintain a cytoskeletal architecture and form actomyosin filaments (López-Fagundo et al., 2014; Poitelon et al., 2016; Urbanski et al., 2016). In addition, the presence of laminin in stiff substrate (40 kPa) affects SC morphology, allowing them to spread, and activate YAP/TAZ mechanotransducers, as characterized by the transfer of YAP and TAZ in SC nucleus (Poitelon et al., 2016; Urbanski et al., 2016). These data show that, in contrast to most anchorage-dependant cells, SCs require laminin to respond to the stiffness of their substrate. The use of static two-dimensional monoculture to study the response of SCs to their environment remains limited. Peripheral nerves are continuously stimulated by stretched movements of the limbs and we do not know how compression, demyelination or loss of axons affects the stiffness of peripheral nerves.

### Advances in Understanding SC Mechanobiology *in Vivo*

The first reports that studied YAP/TAZ in SCs *in vivo* confirmed that both mechanotransducers are active in SCs during axon-SC recognition, while SCs are myelinating and after completion of myelination (Poitelon et al., 2016; Deng et al., 2017; Grove et al., 2017). During development, YAP/TAZ regulates SC proliferation, differentiation and myelination programs (Lopez-Anido et al., 2016; Poitelon et al., 2016; Deng et al., 2017; Grove et al., 2017). SCs ablated for both YAP/TAZ show an arrest of proliferation, fail to associate with an axon and do not initiate myelination (Poitelon et al., 2016; Deng et al., 2017; Grove et al., 2017). The role of YAP/TAZ after myelination remains unclear as reports conflict on their requirements in adult SCs (Deng et al., 2017; Grove et al., 2017). Interestingly, YAP/TAZ regulates in concert several pathways including lipid or sterol biosynthesis in SCs but also integrins (i.e., integrin α6) and G-protein signaling (i.e., Gαs), suggesting respectively positive and negative feedback loops (Poitelon et al., 2016; Deng et al., 2017). YAP and TAZ are functionally redundant, yet it appears that TAZ has a more prominent role in SC development (Poitelon et al., 2016; Deng et al., 2017). Recent findings showed that after nerve injury TAZ levels are increased (Mindos et al., 2017). On the other hand, YAP levels remain stable in control animals and ablation of YAP alone does not affect peripheral nerve remyelination (Mindos et al., 2017). These data hint toward a specific role for TAZ in peripheral nerve remyelination. On the other hand, independent work demonstrated that YAP alone plays a role in the modulation of internodal length and positively regulates myelination and myelin elongation (Fernando et al., 2016). Interestingly, in a model deficient for the laminin receptor Dystroglycan, the amount of active YAP is reduced and internodes are shorter. However upon bone expansion, the peripheral nerves are stretched, the activity of YAP in SCs is increased and myelination appears to be improved (Fernando et al., 2016). These data suggest that physical stretch of the nerve could be a new therapeutic path to improve myelination. It is unclear if the physical stimulation provided by the stretch comes from CAMs at the axon interface, at the ECM interface or both. However, as myelin internodal length is known to grow together with limbs (Thomas and Young, 1949), it will be important to assess if the stretch affects also myelin thickness.

## Using SC Mechanobiology in Therapeutic Approaches for Peripheral Neuropathies

An exceptional quality of SCs is their ability to dedifferentiate from myelinated SCs to become repair SCs. After neurotmesis or axonotmesis, repair SCs are able to remyelinate newly regenerated axons, but most importantly, they provide the necessary tracks for axon guidance and support axonal regeneration. The function of repair SCs is highly considered in peripheral nerves injuries, but also in spinal cord injuries (Xu et al., 1995; Rutkowski et al., 2004; Oudega and Xu, 2006; Kanno et al., 2015). The non-invasive stimulations of SCs at the point of injury, or through the peripheral nerve in peripheral neuropathies, could be an approach to stimulate repair SCs to enhance recovery. The application of pulsed ultrasound on SCs *in vitro* appears to promote SC proliferation and prevent SC differentiation (Zhang et al., 2009; Tsuang et al., 2011). *In vivo* application of ultrasound after axonotmesis, also showed that SCs appeared to be affected, promoting an acceleration of peripheral nerve regeneration (Chang and Hsu, 2004; Raso et al., 2005; Jiang et al., 2016). Contrastingly, a most recent report showed that in SCs alone or in co-culture with neurons, ultrasonic stimulation promotes myelination signaling, with the increase of EGR2 levels in SCs (Yue et al., 2016). Thus it remains indispensable to precisely elucidate the influence of the ultrasound on SCs and peripheral nerves as well as the level and frequency of the ultrasound application. Another approach to stimulate SCs mechanically is the use of electromagnetic fields. Less studied, the stimulation of SCs by electromagnetic fields also opens interesting perspectives in peripheral nerve regeneration (Sisken et al., 1989; Kanje, 1992). Indeed, application of an electromagnetic field to cultured SCs promotes their proliferation and migration, presumably through YAP/TAZ (Colciago et al., 2015). Finally, physical therapy has also been considered as a therapeutic approach to stimulate tensile strain on peripheral nerves. Tensile stimulation, under a certain magnitude, appears to improve the regeneration of peripheral nerves (Bueno and Shah, 2008). However, much of this evidence is empirical and it is unclear how tension strain would stimulate axonal regrowth directly, or indirectly through SCs. In addition, the precise stresses required on a particular injured nerve are difficult to evaluate and could be more damaging. Thus patients could undergo such procedures only once the axon regeneration of nerve is completed (Suszynski et al., 2015).

## Conclusion

SCs are poised to be the target of non-invasive mechanical therapies. Mechanically stimulating SCs could be of interest in traumatic neuropathies and demyelinating peripheral neuropathies, such as Charcot-Marie-Tooth disease, chronic inflammatory demyelinating polyradiculoneuropathy or Guillain-Barré syndrome. Yet, there is first a critical need to better understand SC mechanobiology. Determining the identity of the SC mechanosensors and mechanotransducers will define the signaling pathways and biochemical targets downstream of mechanical stress and help establish multidisciplinary approaches toward remyelination. Such research will also have the potential to be extended to strategies at reducing aberrant proliferation of SCs occurring in Schwannomas or neurofibromas.

## Author Contributions

SB and YP wrote the manuscript; SB, KLZ and YP revised the manuscript; YP created the figures. All authors approved the final version of the manuscript for publication.

## Conflict of Interest Statement

The authors declare that the research was conducted in the absence of any commercial or financial relationships that could be construed as a potential conflict of interest.

## References

[B1] Abe I. Ochiai N. Ichimura H. Tsujino A. Sun J. Hara Y. (2004). Internodes can nearly double in length with gradual elongation of the adult rat sciatic nerve. J. Orthop. Res. 22, 571–577. 10.1016/s0736-0266(03)00218-3 15099637

[B2] Aragona M. Panciera T. Manfrin A. Giulitti S. Michielin F. Elvassore N.. (2013). A mechanical checkpoint controls multicellular growth through YAP/TAZ regulation by actin-processing factors. Cell 154, 1047–1059. 10.1016/j.cell.2013.07.042 23954413

[B3] Baarlink C. Wang H. Grosse R. (2013). Nuclear actin network assembly by formins regulates the SRF coactivator MAL. Science 340, 864–867. 10.1126/science.1235038 23558171

[B4] Balice-Gordon R. J. Bone L. J. Scherer S. S. (1998). Functional gap junctions in the schwann cell myelin sheath. J. Cell Biol. 142, 1095–1104. 10.1083/jcb.142.4.1095 9722620 PMC2132877

[B5] Barres B. A. Chun L. L. Corey D. P. (1990). Ion channels in vertebrate glia. Annu. Rev. Neurosci. 13, 441–474. 10.1146/annurev.neuro.13.1.441 2158266

[B6] Battiston B. Geuna S. Ferrero M. Tos P. (2005). Nerve repair by means of tubulization: literature review and personal clinical experience comparing biological and synthetic conduits for sensory nerve repair. Microsurgery 25, 258–267. 10.1002/micr.20127 15934044

[B7] Beirowski B. (2013). Concepts for regulation of axon integrity by enwrapping glia. Front. Cell. Neurosci. 7:256. 10.3389/fncel.2013.00256 24391540 PMC3867696

[B8] Benninger Y. Thurnherr T. Pereira J. A. Krause S. Wu X. Chrostek-Grashoff A.. (2007). Essential and distinct roles for cdc42 and rac1 in the regulation of Schwann cell biology during peripheral nervous system development. J. Cell Biol. 177, 1051–1061. 10.1083/jcb.200610108 17576798 PMC2064365

[B10] Berti C. Bartesaghi L. Ghidinelli M. Zambroni D. Figlia G. Chen Z. L.. (2011). Non-redundant function of dystroglycan and β1 integrins in radial sorting of axons. Development 138, 4025–4037. 10.1242/dev.065490 21862561 PMC3160097

[B9] Berti C. Nodari A. Wrabetz L. Feltri M. L. (2006). Role of integrins in peripheral nerves and hereditary neuropathies. Neuromolecular Med. 8, 191–204. 10.1385/nmm:8:1:191 16775376

[B11] Boerboom A. Dion V. Chariot A. Franzen R. (2017). Molecular mechanisms involved in schwann cell plasticity. Front. Mol. Neurosci. 10:38. 10.3389/fnmol.2017.00038 28261057 PMC5314106

[B12] Brohawn S. G. Su Z. MacKinnon R. (2014). Mechanosensitivity is mediated directly by the lipid membrane in TRAAK and TREK1 K^+^ channels. Proc. Natl. Acad. Sci. U S A 111, 3614–3619. 10.1073/pnas.1320768111 24550493 PMC3948252

[B13] Brown C. L. Gilbert K. K. Brismee J. M. Sizer P. S. Roger James C. Smith M. P. (2011). The effects of neurodynamic mobilization on fluid dispersion within the tibial nerve at the ankle: an unembalmed cadaveric study. J. Man. Manip. Ther. 19, 26–34. 10.1179/2042618610y.0000000003 22294851 PMC3172954

[B14] Brown R. Pedowitz R. Rydevik B. Woo S. Hargens A. Massie J.. (1993). Effects of acute graded strain on efferent conduction properties in the rabbit tibial nerve. Clin. Orthop. Relat. Res. 296, 288–294. 10.1097/00003086-199311000-00046 8222440

[B15] Brügger V. Engler S. Pereira J. A. Ruff S. Horn M. Welzl H.. (2015). HDAC1/2-dependent P0 expression maintains paranodal and nodal integrity independently of myelin stability through interactions with neurofascins. PLoS Biol. 13:e1002258. 10.1371/journal.pbio.1002258 26406915 PMC4583457

[B16] Bueno F. R. Shah S. B. (2008). Implications of tensile loading for the tissue engineering of nerves. Tissue Eng. Part B Rev. 14, 219–233. 10.1089/ten.teb.2008.0020 18673080

[B19] Bunge M. B. Bunge R. P. Kleitman N. Dean A. C. (1989). Role of peripheral nerve extracellular matrix in Schwann cell function and in neurite regeneration. Dev. Neurosci. 11, 348–360. 10.1159/000111911 2676458

[B17] Bunge M. B. Williams A. K. Wood P. M. (1982). Neuron-Schwann cell interaction in basal lamina formation. Dev. Biol. 92, 449–460. 10.1016/0012-1606(82)90190-7 7117693

[B18] Bunge M. B. Williams A. K. Wood P. M. Uitto J. Jeffrey J. J. (1980). Comparison of nerve cell and nerve cell plus Schwann cell cultures, with particular emphasis on basal lamina and collagen formation. J. Cell Biol. 84, 184–202. 10.1083/jcb.84.1.184 7188611 PMC2110534

[B20] Burnett M. G. Zager E. L. (2004). Pathophysiology of peripheral nerve injury: a brief review. Neurosurg. Focus 16:E1. 10.5772/30060 15174821

[B21] Chang C. J. Hsu S. H. (2004). The effects of low-intensity ultrasound on peripheral nerve regeneration in poly(DL-lactic acid-co-glycolic acid) conduits seeded with Schwann cells. Ultrasound Med. Biol. 30, 1079–1084. 10.1016/j.ultrasmedbio.2004.06.005 15474752

[B22] Chen Y. Wang H. Yoon S. O. Xu X. Hottiger M. O. Svaren J.. (2011). HDAC-mediated deacetylation of NF-κB is critical for Schwann cell myelination. Nat. Neurosci. 14, 437–441. 10.1038/nn.2780 21423191 PMC3074381

[B23] Clarke E. Bearn J. G. (1972). The spiral nerve bands of Fontana. Brain 95, 1–20. 10.1093/brain/95.1.1 4554004

[B24] Codelia V. A. Sun G. Irvine K. D. (2014). Regulation of YAP by mechanical strain through Jnk and Hippo signaling. Curr. Biol. 24, 2012–2017. 10.1016/j.cub.2014.07.034 25127217 PMC4160395

[B25] Colciago A. Melfi S. Giannotti G. Bonalume V. Ballabio M. Caffino L.. (2015). Tumor suppressor Nf2/merlin drives Schwann cell changes following electromagnetic field exposure through Hippo-dependent mechanisms. Cell Death Discov. 1:15021. 10.1038/cddiscovery.2015.21 27551454 PMC4979489

[B26] Colombelli C. Palmisano M. Eshed-Eisenbach Y. Zambroni D. Pavoni E. Ferri C.. (2015). Perlecan is recruited by dystroglycan to nodes of Ranvier and binds the clustering molecule gliomedin. J. Cell Biol. 208, 313–329. 10.1083/jcb.201403111 25646087 PMC4315246

[B27] Coste B. Mathur J. Schmidt M. Earley T. J. Ranade S. Petrus M. J.. (2010). Piezo1 and Piezo2 are essential components of distinct mechanically activated cation channels. Science 330, 55–60. 10.1126/science.1193270 20813920 PMC3062430

[B28] Court F. A. Sherman D. L. Pratt T. Garry E. M. Ribchester R. R. Cottrell D. F.. (2004). Restricted growth of Schwann cells lacking Cajal bands slows conduction in myelinated nerves. Nature 431, 191–195. 10.1038/nature02841 15356632

[B29] Cui Y. Hameed F. M. Yang B. Lee K. Pan C. Q. Park S.. (2015). Cyclic stretching of soft substrates induces spreading and growth. Nat. Commun. 6:6333. 10.1038/ncomms7333 25704457 PMC4346610

[B30] Dahlin L. B. Archer D. R. McLean W. G. (1993). Axonal transport and morphological changes following nerve compression. An experimental study in the rabbit vagus nerve. J. Hand Surg. Br. 18, 106–110. 10.1016/0266-7681(93)90206-u 7679703

[B31] Deng Y. Wu L. M. N. Bai S. Zhao C. Wang H. Wang J.. (2017). A reciprocal regulatory loop between TAZ/YAP and G-protein Gαs regulates Schwann cell proliferation and myelination. Nat. Commun. 8:15161. 10.1038/ncomms15161 28443644 PMC5414202

[B32] Denninger A. R. Breglio A. Maheras K. J. LeDuc G. Cristiglio V. Deme B.. (2015). Claudin-11 tight junctions in myelin are a barrier to diffusion and lack strong adhesive properties. Biophys J. 109, 1387–1397. 10.1016/j.bpj.2015.08.012 26445439 PMC4601091

[B33] Dupont S. Morsut L. Aragona M. Enzo E. Giulitti S. Cordenonsi M.. (2011). Role of YAP/TAZ in mechanotransduction. Nature 474, 179–183. 10.1038/nature10137 21654799

[B34] Dyck P. J. Lais A. C. Giannini C. Engelstad J. K. (1990). Structural alterations of nerve during cuff compression. Proc. Natl. Acad. Sci. U S A 87, 9828–9832. 10.1073/pnas.87.24.9828 2263633 PMC55267

[B35] Earl C. J. Fullerton P. M. Wakefield G. S. Schutta H. S. (1964). Hereditary neuropathy, with liability to pressure palsies; a clinical and electrophysiological study of four families. Q. J. Med. 33, 481–498. 14212604

[B36] Elbediwy A. Vincent-Mistiaen Z. I. Spencer-Dene B. Stone R. K. Boeing S. Wculek S. K.. (2016). Integrin signalling regulates YAP and TAZ to control skin homeostasis. Development 143, 1674–1687. 10.1242/jcs.192518 26989177 PMC4874484

[B37] Esnault C. Stewart A. Gualdrini F. East P. Horswell S. Matthews N.. (2014). Rho-actin signaling to the MRTF coactivators dominates the immediate transcriptional response to serum in fibroblasts. Genes Dev. 28, 943–958. 10.1101/gad.239327.114 24732378 PMC4018493

[B39] Feltri M. L. Graus Porta D. Previtali S. C. Nodari A. Migliavacca B. Cassetti A.. (2002). Conditional disruption of β1 integrin in Schwann cells impedes interactions with axons. J. Cell Biol. 156, 199–209. 10.1083/jcb.200109021 11777940 PMC2173589

[B40] Feltri M. L. Poitelon Y. Previtali S. C. (2015). How schwann cells sort axons: new concepts. Neuroscientist 22, 252–265. 10.1177/1073858415572361 25686621 PMC5181106

[B38] Feltri M. L. Wrabetz L. (2005). Laminins and their receptors in Schwann cells and hereditary neuropathies. J. Peripher. Nerv. Syst. 10, 128–143. 10.1111/j.1085-9489.2005.0010204.x 15958125

[B41] Fernandez-Valle C. Gorman D. Gomez A. M. Bunge M. B. (1997). Actin plays a role in both changes in cell shape and gene-expression associated with Schwann cell myelination. J. Neurosci. 17, 241–250. 8987752 10.1523/JNEUROSCI.17-01-00241.1997 PMC6793673

[B42] Fernando R. N. Cotter L. Perrin-Tricaud C. Berthelot J. Bartolami S. Pereira J. A.. (2016). Optimal myelin elongation relies on YAP activation by axonal growth and inhibition by Crb3/Hippo pathway. Nat. Commun. 7:12186. 10.1038/ncomms12186 27435623 PMC4961766

[B43] Friedland J. C. Lee M. H. Boettiger D. (2009). Mechanically activated integrin switch controls α5β1 function. Science 323, 642–644. 10.1126/science.1168441 19179533

[B44] Fünfschilling U. Supplie L. M. Mahad D. Boretius S. Saab A. S. Edgar J.. (2012). Glycolytic oligodendrocytes maintain myelin and long-term axonal integrity. Nature 485, 517–521. 10.1038/nature11007 22622581 PMC3613737

[B45] Glees P. (1943). Observations on the structure of the connective tissue sheaths of cutaneous nerves. J. Anat. 77, 153–159. 17104922 PMC1252752

[B46] Gray P. T. Bevan S. Ritchie J. M. (1984). High conductance anion-selective channels in rat cultured Schwann cells. Proc. R. Soc. Lond. B Biol. Sci. 221, 395–409. 10.1098/rspb.1984.0041 6146983

[B47] Grove M. Brophy P. J. (2014). FAK is required for Schwann cell spreading on immature basal lamina to coordinate the radial sorting of peripheral axons with myelination. J. Neurosci. 34, 13422–13434. 10.1523/jneurosci.1764-14.2014 25274820 PMC4180476

[B48] Grove M. Kim H. Santerre M. Krupka A. J. Han S. B. Zhai J.. (2017). YAP/TAZ initiate and maintain Schwann cell myelination. Elife 6:e20982. 10.7554/eLife.20982 28124973 PMC5287714

[B49] Grove M. Komiyama N. H. Nave K. A. Grant S. G. Sherman D. L. Brophy P. J. (2007). FAK is required for axonal sorting by Schwann cells. J. Cell Biol. 176, 277–282. 10.1083/jcb.200609021 17242067 PMC2063954

[B50] Gu Y. Ji Y. Zhao Y. Liu Y. Ding F. Gu X.. (2012). The influence of substrate stiffness on the behavior and functions of Schwann cells in culture. Biomaterials 33, 6672–6681. 10.1016/j.biomaterials.2012.06.006 22738780

[B51] Guo J. Wang L. Zhang Y. Wu J. Arpag S. Hu B.. (2014). Abnormal junctions and permeability of myelin in PMP22-deficient nerves. Ann. Neurol. 75, 255–265. 10.1002/ana.24086 24339129 PMC4206215

[B54] Gupta R. Nassiri N. Hazel A. Bathen M. Mozaffar T. (2012). Chronic nerve compression alters Schwann cell myelin architecture in a murine model. Muscle Nerve 45, 231–241. 10.1002/mus.22276 22246880 PMC3262776

[B52] Gupta R. Steward O. (2003). Chronic nerve compression induces concurrent apoptosis and proliferation of Schwann cells. J. Comp. Neurol. 461, 174–186. 10.1002/cne.10692 12724836

[B53] Gupta R. Truong L. Bear D. Chafik D. Modafferi E. Hung C. T. (2005). Shear stress alters the expression of myelin-associated glycoprotein (MAG) and myelin basic protein (MBP) in Schwann cells. J. Orthop. Res. 23, 1232–1239. 10.1016/j.orthres.2004.12.010 16140204

[B55] Hartline D. K. Colman D. R. (2007). Rapid conduction and the evolution of giant axons and myelinated fibers. Curr. Biol. 17, R29–R35. 10.1016/j.cub.2006.11.042 17208176

[B56] Hernandez M. Patzig J. Mayoral S. R. Costa K. D. Chan J. R. Casaccia P. (2016). Mechanostimulation promotes nuclear and epigenetic changes in oligodendrocytes. J. Neurosci. 36, 806–813. 10.1523/JNEUROSCI.2873-15.2016 26791211 PMC4719016

[B57] Hodgson A. J. (1993). Avoiding tourniquet-induced neuropathy through cuff design. Biomed. Instrum. Technol. 27, 401–407. 8220634

[B58] Horowitz S. H. Spollen L. E. Yu W. (2004). Hereditary neuropathy with liability to pressure palsy: fulminant development with axonal loss during military training. J. Neurol. Neurosurg. Psychiatry 75, 1629–1631. 10.1136/jnnp.2003.029314 15489403 PMC1738805

[B59] Hu B. Arpag S. Zhang X. Mobius W. Werner H. Sosinsky G.. (2016). Tuning PAK activity to rescue abnormal myelin permeability in HNPP. PLoS Genet. 12:e1006290. 10.1371/journal.pgen.1006290 27583434 PMC5008806

[B60] Humphrey J. D. Dufresne E. R. Schwartz M. A. (2014). Mechanotransduction and extracellular matrix homeostasis. Nat. Rev. Mol. Cell Biol. 15, 802–812. 10.1038/nrm3896 25355505 PMC4513363

[B61] Ichimura H. Shiga T. Abe I. Hara Y. Terui N. Tsujino A.. (2005). Distribution of sodium channels during nerve elongation in rat peripheral nerve. J. Orthop. Sci. 10, 214–220. 10.1007/s00776-004-0870-8 15815871

[B62] Isermann P. Lammerding J. (2013). Nuclear mechanics and mechanotransduction in health and disease. Curr. Biol. 23, R1113–R1121. 10.1016/j.cub.2013.11.009 24355792 PMC3883624

[B63] Jaalouk D. E. Lammerding J. (2009). Mechanotransduction gone awry. Nat. Rev. Mol. Cell Biol. 10, 63–73. 10.1038/nrm2597 19197333 PMC2668954

[B64] Jacob C. Christen C. N. Pereira J. A. Somandin C. Baggiolini A. Lotscher P.. (2011). HDAC1 and HDAC2 control the transcriptional program of myelination and the survival of Schwann cells. Nat. Neurosci. 14, 429–436. 10.1038/nn.2762 21423190

[B65] Jansen K. A. Donato D. M. Balcioglu H. E. Schmidt T. Danen E. H. Koenderink G. H. (2015). A guide to mechanobiology: where biology and physics meet. Biochim. Biophys. Acta 1853, 3043–3052. 10.1016/j.bbamcr.2015.05.007 25997671

[B66] Jessen K. R. Mirsky R. (2016). The repair Schwann cell and its function in regenerating nerves. J. Physiol. 594, 3521–3531. 10.1113/JP270874 26864683 PMC4929314

[B67] Jiang W. Wang Y. Tang J. Peng J. Wang Y. Guo Q.. (2016). Low-intensity pulsed ultrasound treatment improved the rate of autograft peripheral nerve regeneration in rat. Sci. Rep. 6:22773. 10.1038/srep22773 27102358 PMC4840319

[B68] Jin F. Dong B. Georgiou J. Jiang Q. Zhang J. Bharioke A.. (2011). N-WASp is required for Schwann cell cytoskeletal dynamics, normal myelin gene expression and peripheral nerve myelination. Development 138, 1329–1337. 10.1242/dev.058677 21385763 PMC3188810

[B69] Jou I. M. Lai K. A. Shen C. L. Yamano Y. (2000). Changes in conduction, blood flow, histology, and neurological status following acute nerve-stretch injury induced by femoral lengthening. J. Orthop. Res. 18, 149–155. 10.1002/jor.1100180121 10716291

[B70] Kanje M. (1992). Regeneration of an adult peripheral nerve preparation in culture. Mol. Neurobiol. 6, 217–223. 10.1007/bf02780554 1282333

[B71] Kanno H. Pearse D. D. Ozawa H. Itoi E. Bunge M. B. (2015). Schwann cell transplantation for spinal cord injury repair: its significant therapeutic potential and prospectus. Rev. Neurosci. 26, 121–128. 10.1515/revneuro-2014-0068 25581750

[B72] Kidd G. J. Ohno N. Trapp B. D. (2013). Biology of Schwann cells. Handb. Clin. Neurol. 115, 55–79. 10.1016/B978-0-444-52902-2.00005-9 23931775

[B73] Kim N. G. Gumbiner B. M. (2015). Adhesion to fibronectin regulates Hippo signaling via the FAK-Src-PI3K pathway. J. Cell Biol. 210, 503–515. 10.1083/jcb.201501025 26216901 PMC4523609

[B74] Kong F. Garcia A. J. Mould A. P. Humphries M. J. Zhu C. (2009). Demonstration of catch bonds between an integrin and its ligand. J. Cell Biol. 185, 1275–1284. 10.1083/jcb.200810002 19564406 PMC2712956

[B75] Kwan M. K. Wall E. J. Massie J. Garfin S. R. (1992). Strain, stress and stretch of peripheral nerve. Rabbit experiments *in vitro* and *in vivo*. Acta Orthop. Scand. 63, 267–272. 10.3109/17453679209154780 1609588

[B77] Lee Y. Morrison B. M. Li Y. Lengacher S. Farah M. H. Hoffman P. N.. (2012). Oligodendroglia metabolically support axons and contribute to neurodegeneration. Nature 487, 443–448. 10.1038/nature11314 22801498 PMC3408792

[B76] Lee S. K. Wolfe S. W. (2000). Peripheral nerve injury and repair. J. Am. Acad. Orthop. Surg. 8, 243–252. 10.5435/00124635-200007000-00005 10951113

[B78] Leitman E. M. Tewari A. Horn M. Urbanski M. Damanakis E. Einheber S.. (2011). MLCK regulates Schwann cell cytoskeletal organization, differentiation and myelination. J. Cell Sci. 124, 3784–3796. 10.1242/jcs.080200 22100921 PMC3225267

[B79] Li J. (2015). Molecular regulators of nerve conduction—Lessons from inherited neuropathies and rodent genetic models. Exp. Neurol. 267, 209–218. 10.1016/j.expneurol.2015.03.009 25792482 PMC4417062

[B80] Litvinov R. I. Barsegov V. Schissler A. J. Fisher A. R. Bennett J. S. Weisel J. W.. (2011). Dissociation of bimolecular αIIbβ3-fibrinogen complex under a constant tensile force. Biophys J. 100, 165–173. 10.1016/j.bpj.2010.11.019 21190668 PMC3010843

[B81] Lopez-Anido C. Poitelon Y. Gopinath C. Moran J. J. Ma K. H. Law W. D.. (2016). Tead1 regulates the expression of Peripheral Myelin Protein 22 during Schwann cell development. Hum. Mol. Genet. 25, 3055–3069. 10.1093/hmg/ddw158 27288457 PMC5181599

[B82] López-Fagundo C. Bar-Kochba E. Livi L. L. Hoffman-Kim D. Franck C. (2014). Three-dimensional traction forces of Schwann cells on compliant substrates. J. R. Soc. Interface 11:20140247. 10.1098/rsif.2014.0247 24872498 PMC4208357

[B83] Love J. M. Bober B. G. Orozco E. White A. T. Bremner S. N. Lovering R. M.. (2017). mTOR regulates peripheral nerve response to tensile strain. J. Neurophysiol. 117, 2075–2084. 10.1152/jn.00257.2016 28250148 PMC5434482

[B84] Lundborg G. Rydevik B. (1973). Effects of stretching the tibial nerve of the rabbit. A preliminary study of the intraneural circulation and the barrier function of the perineurium. J. Bone Joint Surg. Br. 55, 390–401. 4707307

[B85] Mackinnon S. E. Dellon A. L. Hudson A. R. Hunter D. A. (1985). A primate model for chronic nerve compression. J. Reconstr. Microsurg. 1, 185–195. 10.1055/s-2007-1007073 4057158

[B86] Masaki T. (2012). Polarization and myelination in myelinating glia. ISRN Neurol. 2012:769412. 10.5402/2012/769412 23326681 PMC3544266

[B87] Maxwell W. L. Irvine A. Graham Adams J. H. Gennarelli T. A. Tipperman R.. (1991). Focal axonal injury: the early axonal response to stretch. J. Neurocytol. 20, 157–164. 10.1007/bf01186989 1709964

[B88] Melendez-Vasquez C. V. Einheber S. Salzer J. L. (2004). Rho kinase regulates schwann cell myelination and formation of associated axonal domains. J. Neurosci. 24, 3953–3963. 10.1523/JNEUROSCI.4920-03.2004 15102911 PMC6729425

[B89] Merolli A. Mingarelli L. Rocchi L. (2012). A more detailed mechanism to explain the “bands of Fontana” in peripheral nerves. Muscle Nerve 46, 540–547. 10.1002/mus.23422 22987695

[B90] Mindos T. Dun X. P. North K. Doddrell R. D. Schulz A. Edwards P.. (2017). Merlin controls the repair capacity of Schwann cells after injury by regulating Hippo/YAP activity. J. Cell Biol. 216, 495–510. 10.1083/jcb.201606052 28137778 PMC5294779

[B91] Miyamoto T. Morita K. Takemoto D. Takeuchi K. Kitano Y. Miyakawa T.. (2005). Tight junctions in Schwann cells of peripheral myelinated axons: a lesson from claudin-19-deficient mice. J. Cell Biol. 169, 527–538. 10.1083/jcb.200501154 15883201 PMC2171943

[B92] Mohseni M. Sun J. Lau A. Curtis S. Goldsmith J. Fox V. L.. (2014). A genetic screen identifies an LKB1-MARK signalling axis controlling the Hippo-YAP pathway. Nat. Cell Biol. 16, 108–117. 10.1038/ncb2884 24362629 PMC4159053

[B93] Monk K. R. Feltri M. L. Taveggia C. (2015). New insights on Schwann cell development. Glia 63, 1376–1393. 10.1002/glia.22852 25921593 PMC4470834

[B94] Montani L. Buerki-Thurnherr T. de Faria J. P. Pereira J. A. Dias N. G. Fernandes R.. (2014). Profilin 1 is required for peripheral nervous system myelination. Development 141, 1553–1561. 10.1242/dev.101840 24598164

[B95] Morell P. Quarles R. H. (1999). “The myelin sheath,” in Basic Neurochemistry: Molecular, Cellular, and Medical Aspects, ed. Siegel G. J. (Philadelphia, PA: Lippincott Williams and Wilkins), 1183.

[B96] Myers R. R. Powell H. C. Costello M. L. Lampert P. W. Zweifach B. W. (1978). Endoneurial fluid pressure: direct measurement with micropipettes. Brain Res. 148, 510–515. 10.1016/0006-8993(78)90739-4 656947

[B97] Nodari A. Zambroni D. Quattrini A. Court F. A. D’Urso A. Recchia A.. (2007). β1 integrin activates Rac1 in Schwann cells to generate radial lamellae during axonal sorting and myelination. J. Cell Biol. 177, 1063–1075. 10.1083/jcb.200610014 17576799 PMC2064366

[B98] Novak N. Bar V. Sabanay H. Frechter S. Jaegle M. Snapper S. B.. (2011). N-WASP is required for membrane wrapping and myelination by Schwann cells. J. Cell Biol. 192, 243–250. 10.1083/jcb.201010013 21263026 PMC3172181

[B99] O’Brien J. P. Mackinnon S. E. MacLean A. R. Hudson A. R. Dellon A. L. Hunter D. A. (1987). A model of chronic nerve compression in the rat. Ann. Plast. Surg. 19, 430–435. 10.1097/00000637-198711000-00008 3688790

[B100] Ochoa J. Danta G. Fowler T. J. Gilliatt R. W. (1971). Nature of the nerve lesion caused by a pneumatic tourniquet. Nature 233, 265–266. 10.1038/233265a0 4999642

[B101] Ochoa J. Fowler T. J. Gilliatt R. W. (1972). Anatomical changes in peripheral nerves compressed by a pneumatic tourniquet. J. Anat. 113, 433–455. 4197303 PMC1271414

[B102] Ohler B. Graf K. Bragg R. Lemons T. Coe R. Genain C.. (2004). Role of lipid interactions in autoimmune demyelination. Biochim. Biophys. Acta 1688, 10–17. 10.1016/j.bbadis.2003.10.001 14732476

[B103] Olson E. N. Nordheim A. (2010). Linking actin dynamics and gene transcription to drive cellular motile functions. Nat. Rev. Mol. Cell Biol. 11, 353–365. 10.1038/nrm2890 20414257 PMC3073350

[B104] Oudega M. Xu X. M. (2006). Schwann cell transplantation for repair of the adult spinal cord. J. Neurotrauma 23, 453–467. 10.1089/neu.2006.23.453 16629629

[B105] Peltonen S. Alanne M. Peltonen J. (2013). Barriers of the peripheral nerve. Tissue Barriers 1:e24956. 10.4161/tisb.24956 24665400 PMC3867511

[B106] Pereira J. A. Benninger Y. Baumann R. Goncalves A. F. Ozcelik M. Thurnherr T.. (2009). Integrin-linked kinase is required for radial sorting of axons and Schwann cell remyelination in the peripheral nervous system. J. Cell Biol. 185, 147–161. 10.1083/jcb.200809008 19349584 PMC2700520

[B107] Pham K. Nassiri N. Gupta R. (2009). c-Jun, krox-20 and integrin β4 expression following chronic nerve compression injury. Neurosci. Lett. 465, 194–198. 10.1016/j.neulet.2009.09.014 19765400 PMC3262774

[B108] Phillips J. B. Smit X. De Zoysa N. Afoke A. Brown R. A. (2004). Peripheral nerves in the rat exhibit localized heterogeneity of tensile properties during limb movement. J. Physiol. 557, 879–887. 10.1113/jphysiol.2004.061804 15064329 PMC1665165

[B109] Plessner M. Melak M. Chinchilla P. Baarlink C. Grosse R. (2015). Nuclear F-actin formation and reorganization upon cell spreading. J. Biol. Chem. 290, 11209–11216. 10.1074/jbc.M114.627166 25759381 PMC4416828

[B111] Poitelon Y. Bogni S. Matafora V. Della-Flora Nunes G. Hurley E. Ghidinelli M.. (2015). Spatial mapping of juxtacrine axo-glial interactions identifies novel molecules in peripheral myelination. Nat. Commun. 6:8303. 10.1038/ncomms9303 26383514 PMC4576721

[B110] Poitelon Y. Lopez-Anido C. Catignas K. Berti C. Palmisano M. Williamson C.. (2016). YAP and TAZ control peripheral myelination and the expression of laminin receptors in Schwann cells. Nat. Neurosci. 19, 879–887. 10.1038/nn.4316 27273766 PMC4925303

[B113] Poliak S. Matlis S. Ullmer C. Scherer S. S. Peles E. (2002). Distinct claudins and associated PDZ proteins form different autotypic tight junctions in myelinating Schwann cells. J. Cell Biol. 159, 361–372. 10.1083/jcb.200207050 12403818 PMC2173042

[B112] Poliak S. Peles E. (2003). The local differentiation of myelinated axons at nodes of Ranvier. Nat. Rev. Neurosci. 4, 968–980. 10.1038/nrn1253 14682359

[B114] Pourmand R. Ochs S. Jersild R. A. Jr. (1994). The relation of the beading of myelinated nerve fibers to the bands of Fontana. Neuroscience 61, 373–380. 10.1016/0306-4522(94)90238-0 7969916

[B115] Power B. J. O’Reilly G. Murphy R. Murphy K. J. Pickering M. Jones J. F. (2015). Normal nerve striations are altered in the trembler-J mouse, a model of Charcot-Marie-Tooth disease. Muscle Nerve 51, 246–252. 10.1002/mus.24303 24890015

[B116] Ranade S. S. Syeda R. Patapoutian A. (2015). Mechanically activated ion channels. Neuron 87, 1162–1179. 10.1016/j.neuron.2015.08.032 26402601 PMC4582600

[B117] Raso V. V. Barbieri C. H. Mazzer N. Fasan V. S. (2005). Can therapeutic ultrasound influence the regeneration of peripheral nerves? J. Neurosci. Methods 142, 185–192. 10.1016/j.jneumeth.2004.08.016 15698658

[B118] Rempel D. Dahlin L. Lundborg G. (1999). Pathophysiology of nerve compression syndromes: response of peripheral nerves to loading. J. Bone Joint Surg. Am. 81, 1600–1610. 10.2106/00004623-199911000-00013 10565653

[B119] Rosenbluth J. (2005). “Glial membranes and axoglial junctions,” in Neuroglia, eds Kettenmann H. Ransom B. R. (New York, NY: Oxford University Press), 601.

[B120] Rosso G. Liashkovich I. Gess B. Young P. Kun A. Shahin V. (2014). Unravelling crucial biomechanical resilience of myelinated peripheral nerve fibres provided by the Schwann cell basal lamina and PMP22. Sci. Rep. 4:7286. 10.1038/srep07286 25446378 PMC4250911

[B121] Rutkowski G. E. Miller C. A. Jeftinija S. Mallapragada S. K. (2004). Synergistic effects of micropatterned biodegradable conduits and Schwann cells on sciatic nerve regeneration. J. Neural Eng. 1, 151–157. 10.1088/1741-2560/1/3/004 15876634

[B122] Rydevik B. L. Kwan M. K. Myers R. R. Brown R. A. Triggs K. J. Woo S. L.. (1990). An *in vitro* mechanical and histological study of acute stretching on rabbit tibial nerve. J. Orthop. Res. 8, 694–701. 10.1002/jor.1100080511 2388109

[B123] Salzer J. L. (2015). Schwann cell myelination. Nature 7:a020529. 10.1101/cshperspect.a020529 26054742 PMC4526746

[B124] Salzer J. L. Bunge R. P. (1980). Studies of Schwann cell proliferation. I. An analysis in tissue culture of proliferation during development, Wallerian degeneration, and direct injury. J. Cell Biol. 84, 739–752. 10.1083/jcb.84.3.739 6244318 PMC2110577

[B125] Salzer J. L. Brophy P. J. Peles E. (2008). Molecular domains of myelinated axons in the peripheral nervous system. Glia 56, 1532–1540. 10.1002/glia.20750 18803321

[B126] Seddon H. J. Medawar P. B. Smith H. (1943). Rate of regeneration of peripheral nerves in man. J. Physiol. 102, 191–215. 10.1113/jphysiol.1943.sp004027 16991601 PMC1393392

[B127] Shaharabani R. Ram-On M. Avinery R. Aharoni R. Arnon R. Talmon Y.. (2016). Structural transition in myelin membrane as initiator of multiple sclerosis. J. Am. Chem. Soc. 138, 12159–12165. 10.1021/jacs.6b04826 27548321

[B128] Simpson A. H. Gillingwater T. H. Anderson H. Cottrell D. Sherman D. L. Ribchester R. R.. (2013). Effect of limb lengthening on internodal length and conduction velocity of peripheral nerve. J. Neurosci. 33, 4536–4539. 10.1523/JNEUROSCI.4176-12.2013 23467369 PMC4335134

[B129] Sisken B. F. Kanje M. Lundborg G. Herbst E. Kurtz W. (1989). Stimulation of rat sciatic nerve regeneration with pulsed electromagnetic fields. Brain Res. 485, 309–316. 10.1016/0006-8993(89)90575-1 2497929

[B130] Small J. R. Ghabriel M. N. Allt G. (1987). The development of Schmidt-Lanterman incisures: an electron microscope study. J. Anat. 150, 277–286. 3654340 PMC1261681

[B131] Sunderland S. (1965). The connective tissues of peripheral nerves. Brain 88, 841–854. 10.1093/brain/88.4.841 4285460

[B132] Sunderland S. (1990). The anatomy and physiology of nerve injury. Muscle Nerve 13, 771–784. 10.1002/mus.880130903 2233864

[B133] Suszynski K. Marcol W. Gorka D. (2015). Physiotherapeutic techniques used in the management of patients with peripheral nerve injuries. Neural Regen. Res. 10, 1770–1772. 10.4103/1673-5374.170299 26807111 PMC4705788

[B134] Tassler P. L. Dellon A. L. Canoun C. (1994). Identification of elastic fibres in the peripheral nerve. J. Hand Surg. Br. 19, 48–54. 10.1016/0266-7681(94)90049-3 8169479

[B135] Taveggia C. Zanazzi G. Petrylak A. Yano H. Rosenbluth J. Einheber S.. (2005). Neuregulin-1 type III determines the ensheathment fate of axons. Neuron 47, 681–694. 10.1016/j.neuron.2005.08.017 16129398 PMC2387056

[B136] Thomas P. K. Jones D. G. (1967). The cellular response to nerve injury. II. Regeneration of the perineurium after nerve section. J. Anat. 101, 45–55. 6047702 PMC1270857

[B137] Thomas P. K. Young J. Z. (1949). Internode lengths in the nerves of fishes. J. Anat. 83, 336–350. 15407240 PMC1273083

[B138] Tsuang Y. H. Liao L. W. Chao Y. H. Sun J. S. Cheng C. K. Chen M. H.. (2011). Effects of low intensity pulsed ultrasound on rat Schwann cells metabolism. Artif. Organs 35, 373–383. 10.1111/j.1525-1594.2010.01086.x 20946299

[B139] Urbanski M. M. Kingsbury L. Moussouros D. Kassim I. Mehjabeen S. Paknejad N.. (2016). Myelinating glia differentiation is regulated by extracellular matrix elasticity. Sci. Rep. 6:33751. 10.1038/srep33751 27646171 PMC5028715

[B140] Viader A. Golden J. P. Baloh R. H. Schmidt R. E. Hunter D. A. Milbrandt J. (2011). Schwann cell mitochondrial metabolism supports long-term axonal survival and peripheral nerve function. J. Neurosci. 31, 10128–10140. 10.1523/JNEUROSCI.0884-11.2011 21752989 PMC3147283

[B141] Vizoso A. D. Young J. Z. (1948). Internode length and fibre diameter in developing and regenerating nerves. J. Anat. 82, 110–134. 17105043 PMC1272963

[B143] Wall E. J. Kwan M. K. Rydevik B. L. Woo S. L. Garfin S. R. (1991). Stress relaxation of a peripheral nerve. J. Hand Surg. Am. 16, 859–863. 10.1016/S0363-5023(10)80149-2 1940164

[B142] Wall E. J. Massie J. B. Kwan M. K. Rydevik B. L. Myers R. R. Garfin S. R. (1992). Experimental stretch neuropathy. Changes in nerve conduction under tension. J. Bone Joint Surg. Br. 74, 126–129. 1732240 10.1302/0301-620X.74B1.1732240

[B144] Wang H. Tewari A. Einheber S. Salzer J. L. Melendez-Vasquez C. V. (2008). Myosin II has distinct functions in PNS and CNS myelin sheath formation. J. Cell Biol. 182, 1171–1184. 10.1083/jcb.200802091 18794332 PMC2542477

[B145] Wu L. M. Wang J. Conidi A. Zhao C. Wang H. Ford Z.. (2016). Zeb2 recruits HDAC-NuRD to inhibit Notch and controls Schwann cell differentiation and remyelination. Nat. Neurosci. 19, 1060–1072. 10.1038/nn.4322 27294509 PMC4961522

[B146] Xu X. M. Guénard V. Kleitman N. Bunge M. B. (1995). Axonal regeneration into Schwann cell-seeded guidance channels grafted into transected adult rat spinal cord. J. Comp. Neurol. 351, 145–160. 10.1002/cne.903510113 7896937

[B147] Yin X. Kidd G. J. Nave K. A. Trapp B. D. (2008). P0 protein is required for and can induce formation of schmidt-lantermann incisures in myelin internodes. J. Neurosci. 28, 7068–7073. 10.1523/JNEUROSCI.0771-08.2008 18614675 PMC2682947

[B148] Yin X. Kiryu-Seo S. Kidd G. J. Feltri M. L. Wrabetz L. Trapp B. D. (2015). Proteolipid protein cannot replace P0 protein as the major structural protein of peripheral nervous system myelin. Glia 63, 66–77. 10.1002/glia.22733 25066805 PMC4237650

[B149] Yue Y. Yang X. Zhang L. Xiao X. Nabar N. R. Lin Y.. (2016). Low-intensity pulsed ultrasound upregulates pro-myelination indicators of Schwann cells enhanced by co-culture with adipose-derived stem cells. Cell Prolif. 49, 720–728. 10.1111/cpr.12298 27625295 PMC6496622

[B150] Zachary L. S. Dellon E. S. Nicholas E. M. Dellon A. L. (1993). The structural basis of Felice Fontana’s spiral bands and their relationship to nerve injury. J. Reconstr. Microsurg. 9, 131–138. 10.1055/s-2007-1006661 8468703

[B151] Zalc B. Colman D. R. (2000). Origins of vertebrate success. Science 288, 271–272. 10.1126/science.288.5464.271c 10777405

[B152] Zalc B. Goujet D. Colman D. (2008). The origin of the myelination program in vertebrates. Curr. Biol. 18, R511–R512. 10.1016/j.cub.2008.04.010 18579089

[B153] Zhang H. Lin X. Wan H. Li J. H. Li J. M. (2009). Effect of low-intensity pulsed ultrasound on the expression of neurotrophin-3 and brain-derived neurotrophic factor in cultured Schwann cells. Microsurgery 29, 479–485. 10.1002/micr.20644 19308950

